# Regucalcin enhances adipocyte differentiation and attenuates inflammation in 3T3‐L1 cells

**DOI:** 10.1002/2211-5463.12947

**Published:** 2020-08-30

**Authors:** Tomiyasu Murata, Masayoshi Yamaguchi, Susumu Kohno, Chiaki Takahashi, Watanabe Risa, Kanna Hatori, Kiyomi Hikita, Norio Kaneda

**Affiliations:** ^1^ Laboratory of Analytical Neurobiology Faculty of Pharmacy Meijo University Nagoya Japan; ^2^ Cancer Biology Program University of Hawaii Cancer Center University of Hawaii at Manoa Honolulu HI USA; ^3^ Division of Oncology and Molecular Biology Cancer Research Institute Kanazawa University Kanazawa Japan

**Keywords:** 3T3‐L1 cells, adipocyte differentiation, inflammation, RAW264.7 macrophage cells, regucalcin

## Abstract

Dysregulation of adipocyte differentiation and dysfunction play key roles in the pathogenesis of obesity and associated disorders such as diabetes and metabolic syndrome, and as such, a better understanding of the molecular mechanism of adipogenesis may help to elucidate the pathological condition of obesity and its associated disorders. Regucalcin (RGN) plays multiple regulatory roles in intracellular Ca^2+^ signaling pathways in mammalian cells. Here, we report that overexpression of RGN enhances lipid accumulation in 3T3‐L1 adipocyte cells after adipogenic stimulation, accompanied by upregulation of adipocyte differentiation marker proteins. In contrast, genetic disruption of RGN inhibited adipogenic stimulation‐induced differentiation of 3T3‐L1 cells. Furthermore, RGN overexpression in differentiated 3T3‐L1 adipocytes blocked inflammatory crosstalk between 3T3‐L1 adipocytes and RAW264.7 macrophages in a transwell coculture system. Knockdown of RGN expression in cocultured 3T3‐L1 adipocytes enhanced their susceptibility to RAW264.7 macrophage‐mediated inflammation. These results suggest that RGN is required for 3T3‐L1 adipocyte differentiation and that it exerts anti‐inflammatory activity against 3T3‐L1 adipocyte inflammation after coculture with RAW264.7 macrophages. Thus, RGN may be a novel regulator of adipocyte differentiation and act as a suppressor of inflammation in macrophage‐infiltrated adipocyte tissue.

## Graphical abstract

Regucalcin enhances the differentiation of 3T3‐L1 cells into adipocytes. Regucalcin in 3T3‐L1 adipocytes also blocks inflammatory crosstalk between 3T3‐L1 adipocytes and RAW264.7 macrophages in a transwell coculture system. Regucalcin is a novel regulator of adipocyte differentiation and acts as a suppressor of macrophage‐associated adipocyte inflammation.

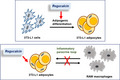

**Abbreviations**
C/EBP: CCAAT enhancer‐binding protein
DMEM: Dulbecco's modified Eagle's medium
FABP4: fatty acid‐binding protein 4
IL‐6: interleukin‐6
MCP‐1: monocyte chemoattractant protein‐1
NF‐κB: nuclear factor‐κB
PPARγ: peroxisome proliferator‐activated receptor γ
RGN: regucalcin
sgRNA: single‐guide RNA
siRNA: small interfering RNA
TNF‐α: tumor necrosis factor‐α

Regucalcin (RGN) plays multiple regulatory roles in intracellular Ca^2+^ signaling pathways in mammalian cells [1, 2]. RGN expression is regulated by various hormonal factors [3, 4, 5]. We previously reported that insulin induces RGN expression in liver cells *in vitro* and *in vivo* [6] and that hepatocyte RGN regulates liver lipid metabolism through insulin action [6]. RGN knockout (KO) mouse studies have shown abnormal glucose tolerance via impairment of insulin secretion [7, 8]. *In vitro* studies using islets of RGN KO mice have shown that deficiency of RGN impairs insulin secretion to glucose or potassium chloride, suggesting the involvement of RGN in insulin secretion in pancreatic β‐cells [7, 9]. Studies on *in vivo* RGN KO mice also revealed that RGN is related to the pathogenesis of diabetic nephropathy, nonalcoholic fatty liver disease, and hepatic steatosis [10, 11]. We also found support for a physiological role of RGN in maintaining the function of kidney proximal tubular epithelial cells [12]. Diabetic nephropathy rats were shown to exhibit downregulated expression of RGN expression in kidney tissue and decreased amounts of RGN‐present exosomes in urine [13].

Adipogenesis is regulated by an elaborate cascade of transcription factors. The peroxisome proliferator‐activated receptor γ (PPARγ) and the members of the CCAAT enhancer‐binding protein (C/EBP) family act as master regulators in the differentiation of preadipocytes [14]. PPARγ transcriptionally regulates the target gene involved in lipid accumulation and metabolism and insulin sensitivity, including fatty acid‐binding protein (FABP4) [15], lipid‐droplet‐associated protein perilipin A [16], and adiponectin [17], leading to adipocyte differentiation. The dysregulation of adipocyte differentiation and dysfunction play key roles in the pathogenesis of obesity and associated disorders such as diabetes and metabolic syndrome [18]. A better understanding of the molecular mechanism of adipogenesis will help to elucidate the pathological condition of obesity and its associated disorders.

Obesity is associated with the infiltration of macrophages into adipocyte tissue [19, 20]. The crosstalk between macrophages and adipocytes triggers and facilitates inflammation in obese adipocyte tissue [19, 20]. This macrophage‐mediated adipocyte inflammation causes obesity‐mediated diseases [21]. Dysregulation of adipokines and chemokines plays an important role in the crosstalk between adipocytes and macrophages and contributes to adipose tissue inflammation [19, 20]. In obesity, proinflammatory mediator tumor necrosis factor‐α (TNF‐α) is one of key mediators of adipose tissue macrophages [22] and contributes to inflammation and insulin resistance in the adipocytes [23]. The inflamed adipocyte itself augments production of various proinflammatory cytokines such as monocyte chemoattractant protein‐1 (MCP‐1), interleukin‐6 (IL‐6), and TNF‐α [24]. MCP‐1 secreted by adipocytes is involved in chronic inflammation by promoting infiltration of macrophages into adipose tissue [25]. Adiponectin is an adipose‐derived anti‐inflammatory factor that is downregulated in obese states [26], and its expression is directly inhibited by TNF‐α in adipocytes [27]. *In vitro* coculture experiments have demonstrated the existence of a paracrine loop between macrophage‐derived TNF‐α and adipocyte‐derived saturated fatty acids [28, 29]. Thus, blocking macrophage‐mediated adipocyte inflammation could be beneficial in preventing obesity and its associated diseases.

Here, we first investigated whether RGN accelerates differentiation of 3T3‐L1 preadipocytes. Our second goal was to investigate whether RGN attenuates TNF‐α‐induced inflammation in 3T3‐L1 adipocytes and blocks inflammatory communication between 3T3‐L1 adipocytes and RAW264.3 macrophages under coculture conditions.

## Materials and methods

### Materials

The following materials from the indicated sources were used in this study: Dulbecco's modified Eagle's medium (DMEM), PBS, FBS, penicillin, streptomycin, Lipofectamine 3000, Lipofectamine RNAiMAX, TRIzol, First Strand cDNA Synthesis Kit, SYBR GreenER qPCR SuperMix, Pierce BCA protein assay reagent kit, and SuperBlock blocking buffer from Thermo Fisher Scientific (Waltham, MA, USA); RNase‐free DNase from Qiagen (Chatsworth, CA, USA); Millicell hanging cell culture inserts (pore size; 0.4 µm; polyethylene terephthalate membrane) for a transwell culture system from Merck Millipore (Darmstadt, Germany); TNF‐α from R&D Systems, Inc. (Minneapolis, MN, USA); insulin, dexamethasone, and 3‐isobutyl‐1‐methylxanthine (IBMX) from Millipore Sigma (St. Louis, MO, USA); and lentiviral vectors containing a single‐guide RNA (sgRNA)/CRISPR/Cas9 All‐in‐One gene targeting system and adenovirus harboring expression of RGN or LacZ from Applied Biological Materials (Richmond, BC, Canada). Also purchased were RGN‐targeting small interfering RNA (siRNA; ON‐TARGETplus SMARTpool) or nontargeting control siRNA (ON‐TARGETplus Nontargeting pool) from Dharmacon (Lafayette, CO, USA); ViroMag R/L viral gene delivery reagent from OZ Biosciences (Marseille, France); Oil Red O staining kit and free fatty acid quantification assay kit from BioVision (Milpitas, CA, USA); free glycerol assay kit from Cell Biolabs (San Diego, CA, USA); triglycerol assay kit from Abcam (Cambridge, UK); pGL4.32[*luc2P*/NF‐κB‐RE/Hygro] vector, and luciferase assay kit from Promega (Madison, WI, USA); antibodies against PPARγ, C/EBPα, perilipin A, adiponectin, and β‐actin from Cell Signaling Technology (Danvers, MA, USA); Can Get Signal solution 1 and 2 from TOYOBO (Osaka, Japan); peroxidase‐conjugated secondary antibody and enhanced chemiluminescent (ECL) western blotting detection reagents from GE Healthcare Life Sciences (Amersham, UK); and protease inhibitor cocktail tablets from Roche (Mannheim, Germany).

### Cell culture and treatment

The 3T3‐L1 cells were cultured in DMEM supplemented with 10% heat‐inactivated FBS, 50 units·mL^−1^ penicillin, and 50 μg·mL^−1^ streptomycin in a humidified atmosphere of 5% CO_2_ and 95% air. The cells were cultured for 2 days to confluence (day 0), and adipogenic differentiation was induced by treatment with DMEM containing 10% FBS, 2 µg·mL^−1^ insulin, 1 µm dexamethasone, and 0.5 mm IBMX for 2 days. After differentiation induction, the medium was changed to differentiation‐maintenance medium containing 10% FBS and insulin and was replaced every 2 days. The RAW264.7 mouse macrophage cell line was maintained in RPMI 1640 medium containing 10% heat‐inactivated FBS, 50 U·mL^−1^ penicillin, and 50 μg·mL^−1^ streptomycin under a humidified atmosphere of 5% CO_2_ and 95% air at 37 °C.

### Assessment of adipocyte differentiation

For Oil Red O staining, after fixation with 5% formaldehyde in PBS for 30 min and washing three times with PBS, the cells were stained with 1.5% Oil Red O for 1 h. After three washings with PBS, the Oil Red O stain was extracted with 100% isopropanol for 30 min and determined by measuring absorbance at 510 nm. For determination of intracellular triglycerol content, triglycerol was measured using a triglycerol assay kit. Briefly, cells were homogenized in 5% NP‐40 solution, and the homogenized samples were heated at 80 °C to solubilize the triglycerols. Next, the samples were mixed with lipase and the TG reaction mixture. After 1 h of incubation, the sample absorbance was measured at 570 nm using a PerkinElmer microplate spectrofluorometer (EnSpire, Norwalk, CT, USA). The triglycerol content was normalized to that of total protein, which was determined using a micro‐BCA protein assay reagent kit.

### Quantitative RT‐PCR analysis

Total RNA was prepared from cultured cells using TRIzol reagent according to the manufacturer's instructions and then treated with RNase‐free DNase. The cDNA was synthesized from total RNA using a high‐capacity cDNA synthesis kit. Quantitative PCR was carried out in a reaction mixture composed of cDNA, specific sense and antisense primers, and SYBR GreenER qPCR SuperMix with LightCycler 480 System II (Roche, Basel, Switzerland). The relative quantity of target mRNA was calculated using the comparative cycle threshold (Ct) method and was normalized using β‐actin as an endogenous control. Nucleotide sequences of the specific primers used were as follows: 5′‐CCTCCCTCTCATCAGTTCTAT‐3′ (sense) and 5′‐ACCAAGTGGAGGAGCAGCTGG‐3′ (antisense) for TNF‐α; 5′‐CCAAACTGGATATAATCAGGA‐3′ (sense) and 5′‐AACCAAGAGGTAAAAGATTTA‐3′ (antisense) for IL‐6; 5′‐CCACGTGTTGGCTCAGCCAGA‐3′ (sense) and 5′‐TTTGTCACCAAGCTCAAGAG‐3′ (antisense) for MCP‐1; 5′‐TGGCAGGCATCCCAGGACATC‐3′ (sense) and 5′‐AAGCCCCGTGGCCCTTCAGCT‐3′ (antisense) for adiponectin; and 5′‐GTGGGCCGCCCTAGGCACCA‐3′ (sense) and 5′‐GGTTGGCCTTAGGGTTCAGG‐3′ (antisense) for β‐actin.

### Immunoblot analysis

Cells were lysed in RIPA lysis buffer containing protease inhibitor mixture. Lysed cells were then centrifuged at 15 000 ***g*** for 15 min at 4 °C, and the supernatants were subjected to western blot analysis. Supernatant proteins were resolved using sodium dodecyl sulfate–polyacrylamide gel electrophoresis and then transferred to polyvinylidene difluoride membranes. After blocking for 1 h in SuperBlock blocking buffer, the membranes were incubated with primary antibody in Can Get Signal solution 1 and then with peroxidase‐conjugated secondary antibody in Can Get Signal solution 2. Bound antibody was visualized using the ECL system. Blots were then probed with anti‐β‐actin antibody to use β‐actin as a loading control.

### Virus‐mediated overexpression of RGN

For preparation of stably RGN‐overexpressing 3T3‐L1 cells, we generated a retrovirus harboring expression of RGN or LacZ using the previously described method [30]. The 3T3‐L1 cells were infected with a retrovirus harboring expression of RGN or LacZ using ViroMag R/L viral gene delivery reagent and then selected with puromycin for 3 weeks and used for the adipocyte differentiation experiment. In transient RGN overexpression experiments of differentiated 3T3‐L1 adipocytes, after 8 days of differentiation induction, the 3T3‐L1 adipocytes were infected with an adenovirus harboring expression of LacZ or RGN at the optimized multiplicity of infection. After 48 h, the infected cells were used for the adipocyte inflammation experiment.

### Virus‐mediated knockout of RGN

For preparation of RGN‐deficient 3T3‐L1 cells, we performed CRISPR/Cas9/sgRNA procedures by using lentiviruses harboring sgRNA‐targeting RGN. The sgRNA sequences targeting RGN were 5′‐TTACGGGAGAACTACAGGTG‐3′, 5′‐TGTGACGCTTCCTCCCATAC‐3′, and 5′‐TCAAGTGCAGCGAGTTGCTG‐3′, and a scrambled sgRNA was used as a control sgRNA. A mixture of three types of lentivirus harboring sgRNA‐targeting RGN was used to transduce 3T3‐L1 cells using ViroMag R/L viral gene delivery reagent and then selected with puromycin for 3 weeks. The RGN KO efficiency was analyzed by western blotting.

### siRNA‐mediated knockdown of RGN

The 3T3‐L1 cells were differentiated into adipocytes until day 8. Then, mature 3T3‐L1 adipocytes were trypsinized and replated in 12‐well or 24‐well plates. Four hours later, transfection with RGN‐targeting siRNA (ON‐TARGETplus SMARTpool) or nontargeting control siRNA (ON‐TARGETplus nontargeting pool) was performed using Lipofectamine RNAiMAX transfection reagent according to the manufacturer's protocol. After 48 h of transfection, the silencing of RGN expression was analyzed by western blotting.

### Determination of lipolysis

Lipolysis was estimated by quantification of the amount of free fatty acids (FFAs) and glycerol into culture medium. Briefly, after treatment, 3T3‐L1 adipocytes were incubated in serum‐free DMEM containing 2% FFA‐free BSA for 12 h. Next, the 3T3‐L1 adipocytes were treated with 10 ng·mL^−1^ TNF‐α or cocultured with RAW264.7 macrophage cells in serum‐free DMEM containing 2% FFA‐free BSA for 24 h. The culture medium was collected, and the amount of FFAs and glycerol was measured using FFA quantification kit and free glycerol assay kit, respectively, according to the manufacturer's instructions. The amount of FFA and glycerol was normalized to that of total protein, which was determined using a micro‐BCA protein assay reagent kit.

### 3T3‐L1 adiocytes–RAW264.7 coculture

To simulate the microenvironment between adipocytes and macrophages, differentiated 3T3‐L1 adipocytes and RAW264.7 macrophage cells were cocultured in a transwell culture system. Differentiated 3T3‐L1 adipocytes were seeded in the bottom of a 6‐ or 12‐well plate. RAW264.7 cells were seeded into 6‐ or 12‐well culture inserts. After pretreatment of differentiated 3T3‐L1 adipocytes, RAW264.7 cells were in turn cocultured with differentiated 3T3‐L1 adipocytes by transferring the culture inserts into a 6‐ or 12‐well plates containing differentiated 3T3‐L1 adipocytes. After the coculture experiment, the RAW264.7 cell culture inserts were removed, and the differentiated 3T3‐L1 adipocytes or RAW264.7 macrophage cells were used for subsequent assay.

### Assay of transcriptional activity of nuclear factor‐κB

The transcriptional activity of nuclear factor‐κB (NF‐κB) was determined by reporter gene assay. Briefly, 3T3‐L1 cells or RAW264.7 macrophages were transfected with the NF‐κB reporter vector (pGL4.32[*luc2P*/NF‐κB‐RE/Hygro] vector) using Lipofectamine 3000 reagent. After 24 h, transfected cells were selected by treatment with hygromycin B and then subcloned by limited dilution. Then, to choose the most responsive cells, stably transfected 3T3‐L1 cells were further screened by measurement of TNF‐α‐induced NF‐κB reporter activity, and stably transfected RAW264.7 macrophages were further screened by measurement of palmitate‐induced NF‐κB reporter activity. For measurement of NF‐κB reporter activity, cells were lysed in reporter buffer and the luciferase activity of the lysates was measured using luciferase assay according to the manufacturer's instructions. The luciferase activity was normalized to that of total protein, which was determined using a micro‐BCA protein assay reagent kit.

### Statistical analysis

All data are presented as means ± SE of three independent experiments, in which measurements were made in triplicate. The significance of differences was estimated by Kruskal–Wallis with Dunn's *post hoc* test. A *P‐*value less than 0.05 was considered to be significant. All analyses were carried out using graphpad prism 5.0 (GraphPad Software, San Diego, CA, USA).

## Results and Discussion

### RGN enhances the differentiation of 3T3‐L1 cells into adipocytes

**Fig. 1:**
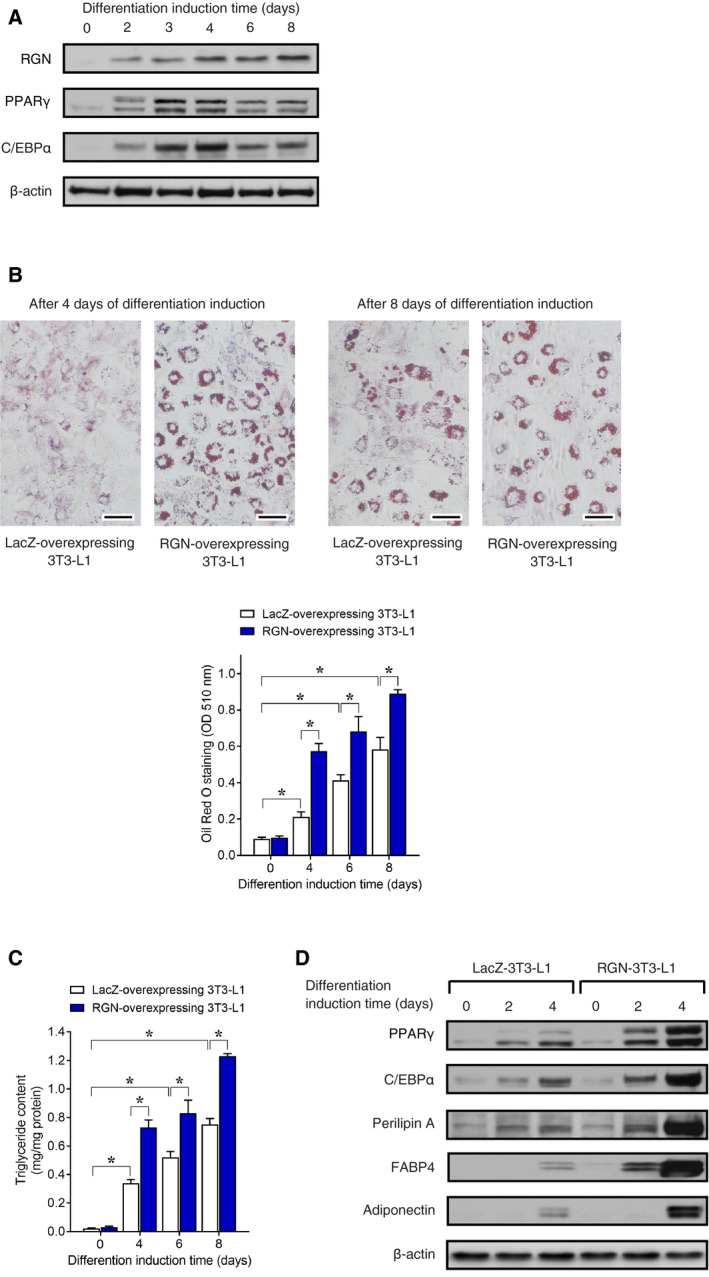
Regucalcin overexpression enhanced adipocyte differentiation of 3T3‐L1 cells. 3T3‐L1 cells overexpressing LacZ or RGN were treated with adipogenic differentiation medium for 2 days and then refreshed with differentiation‐maintenance medium every 2 days until day 8. (A) After the indicated differentiation induction time, cells were lysed and then processed for western blotting analysis using antibodies against RGN, PPARγ, and C/EBPα. β‐Actin was used as a loading control. (B) On the 4th and 8th days after differentiation induction, the cells were stained with Oil Red O and photographed using a microscope. Scale bar = 100 μm. For quantification of lipid accumulation, after differentiation induction, cells were stained with Oil Red O and stained oil droplets were treated with isopropanol to elute Oil Red O dye. The absorbance was measured at 510 nm. Data are presented as means ± SE of three independent experiments performed in triplicate, *P < 0.05 was considered statistically significant. (C) The intracellular triglycerol content was measured using a commercial triglycerol assay kit. Data are presented as means ± SE of three independent experiments performed in triplicate, *P < 0.05 was considered statistically significant. (D) On the second and fourth day after differentiation induction, cells were lysed and then processed for western blotting analysis using antibodies against PPARγ, C/EBPα, perilipin A, FABP4, and adiponectin. β‐Actin was used as a loading control. All data were statistically analyzed using the Kruskal–Wallis and Mann–Whitney *U*‐tests.

To elucidate the functional role of RGN in adipocyte differentiation, we first examined the expression of endogenous RGN protein in 3T3‐L1 cells after differentiation induction (Fig. 1A). The transcription factors PPARγ and C/EBPα are induced at an early stage following adipogenic stimulation and play a central role in adipocyte differentiation [14]. The expression of RGN protein began to increase 2 days after differentiation induction, similar to the expression of PPARγ and C/EBPα, and reached a maximum level on day 4. The maximum expression of RGN expression was continuous by the late stage of differentiation (on day 8). To investigate the effect of RGN overexpression on adipocyte differentiation, we generated LacZ‐ or RGN‐overexpressing 3T3‐L1 cells by retrovirus‐mediated gene transfer and examined whether RGN overexpression altered the adipocyte differentiation of the 3T3‐L1 cells. The 3T3‐L1 cells were stained with Oil Red O to estimate the accumulation of lipid droplets (Fig. 1B). At 4 days after differentiation induction, an increased accumulation of lipid droplets was detected in majority of the RGN‐overexpressing 3T3‐L1 cells, and at 8 days after differentiation induction, the size of the lipid droplets was significantly larger in the RGN‐overexpressing cells than in the LacZ‐overexpressing cells. Following differentiation induction until day 8, lipid accumulation time‐dependently increased in LacZ‐overexpressing 3T3‐L1 cells. Interestingly, RGN overexpression led to significantly higher lipid accumulation relative to the LacZ‐overexpressing 3T3‐L1 cells. In addition, following differentiation induction, the level of triglycerols, a major component of lipid droplets, was also higher in RGN‐overexpressing 3T3‐L1 cells than in LacZ‐overexpressing 3T3‐L1 cells (Fig. 1C). We further examined the effects of RGN overexpression on the expression of adipogenic differentiation markers (Fig. 1D). After 2 and 4 days of differentiation induction, RGN‐overexpressing 3T3‐L1 cells exhibited significantly higher expression of the transcriptional factors PPARγ and C/EBPα in the early stage of differentiation as compared to LacZ‐overexpressing 3T3‐L1 cells. Perilipin A is known to bind to the surface of adipocyte lipid droplets and regulate lipid storage in adipocytes [31]. FABP4 is highly expressed in mature adipocytes and acts as a cytoplasmic fatty acid chaperone [32]. Adiponectin promotes adipocyte differentiation and enhances adipocyte lipid storage [33, 34]. Thus, perilipin A, FABP4, and adiponectin are required for adipocyte maturation. RGN overexpression significantly enhanced the expression of mature adipocyte markers perilipin A, FABP4, and adiponectin after 2 and 4 days of differentiation induction (early time of differentiation) compared with LacZ‐overexpressing 3T3‐L1 cells. Taken together, these results suggest that RGN overexpression enhanced the differentiation of 3T3‐L1 cells into adipocytes.

**Fig. 2:**
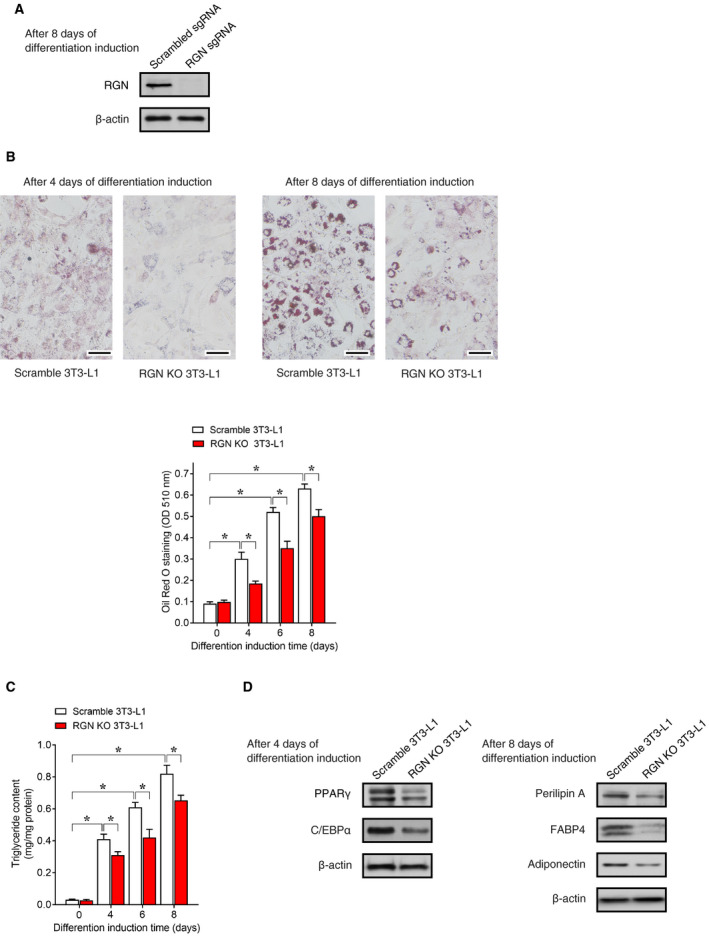
Regucalcin knockout inhibited adipocyte differentiation of 3T3‐L1 cells. Using a lentiviral CRISPR/Cas9 system, scrambled control or RGN knockout (KO) 3T3‐L1 cells were generated. The scrambled control or RGN KO 3T3‐L1 cells were treated with adipogenic differentiation medium for 2 days and then refreshed with differentiation‐maintenance medium every 2 days until day 8. (A) The knockout efficiency of RGN was examined by western blotting using an anti‐RGN antibody. β‐Actin was used as a loading control. (B) Oil Red O staining was performed as described in Fig. 1B. On the 4th and 8th days after differentiation induction, the cells were stained with Oil Red O and photographed using a microscope. Scale bar = 100 μm. Data are presented as means ± SE of three independent experiments performed in triplicate, *P < 0.05 was considered statistically significant. (C) The intracellular triglycerol content was measured using a commercial triglycerol assay kit. Data are presented as means ± SE of three independent experiments performed in triplicate, *P < 0.05 was considered statistically significant. (D) In western blotting, cell lysates after 4 days of differentiation were used for immunoblotting of PPARγ and C/EBPα and cell lysates after 8 days of differentiation were used for immunoblotting of perilipin A, FABP4, and adiponectin. β‐Actin was used as a loading control. All data were statistically analyzed using the Kruskal–Wallis and Mann–Whitney *U*‐tests.

To further investigate the function of RGN in adipogenesis in 3T3‐L1 cells, we used CRISPR/Cas9 to disrupt the RGN gene in 3T3‐L1 cells and then examined the RGN knockout (KO) effect on adipocyte differentiation of 3T3‐L1 cells. As shown in Fig. 2A, the immunoblot analysis of differentiated 3T3‐L1 adipocytes expressing scrambled sgRNA showed obvious expression of endogenous RGN protein, while endogenous RGN protein was not detected in differentiated 3T3‐L1 adipocytes expressing sgRNA‐targeting RGN, indicating successful RGN gene KO in 3T3‐L1 cells. As shown in Fig. 2B, after 4 days of differentiation induction, Oil Red O staining yielded negative results in RGN KO 3T3‐L1 cells, and after 8 days of differentiation, the accumulation of lipid droplets was inhibited by RGN KO. Furthermore, following differentiation induction, the lipid accumulation in the RGN KO 3T3‐L1 cells was significantly lower than in the scrambled control 3T3‐L1 cells until day 8 in a time‐dependent manner (Fig. 2B). The triglycerol content was also lower in RGN KO 3T3‐L1 cells after differentiation induction than in scrambled control 3T3‐L1 cells (Fig. 2C). Furthermore, as shown in Fig. 2D, RGN KO 3T3‐L1 cells exhibited a decrease in expression of PPARγ and C/EBPα on day 4 (early stage of differentiation) and expression of perilipin A, FABP4, and adiponectin on day 8 (late stage of differentiation). Taken together, these suggest that the RGN deficiency impaired adipocyte differentiation of 3T3‐L1 cells by reducing the expression of adipogenic factors. Thus, as in the RGN overexpression experiments, the RGN knockout experiments provided evidence that RGN is essential for differentiation of 3T3‐L1 cells into adipocytes.

It has been demonstrated that mitotic clonal expansion is a prerequisite for adipogenic differentiation in 3T3‐L1 adipocytes [35]. After 2 days of differentiation induction, the proliferation of 3T3‐L1 cells was not altered by RGN overexpression and RGN KO (data not shown), implying that enhancement of adipocyte differentiation by RGN is not dependent on mitotic clonal expansion. In the present study, we found that the expression of endogenous RGN was elevated at the early stage of 3T3‐L1 cell adipogenesis and enhanced adipocyte differentiation of 3T3‐L1 cells, accompanied by upregulation of PPARγ and C/EBPα. PPARγ and C/EBPα are essential transcriptional factors for adipocyte differentiation [14]. We have previously reported that cytoplasmic RGN is translocated into the nucleus of various cell types and plays a multifunctional role in the regulation of nuclear function [36]. Furthermore, the RGN‐mediated nuclear function may regulate the expression of transcription factors responsible for adipogenesis. Because RGN‐deficient 3T3‐L1 cells showed decreased expression of PPARγ and C/EBPα after adipogenic stimulation, RGN might initiate adipocyte differentiation by regulating the adipogenic transcription factor network. In addition, because endogenous RGN expression was observed not only in the early stage but also in the late stage of adipogenic differentiation, RGN also might play a key role in the adipogenic program for maturation of adipocytes.

### RGN attenuates TNF‐α‐induced inflammatory responses in 3T3‐L1 adipocytes

**Fig. 3:**
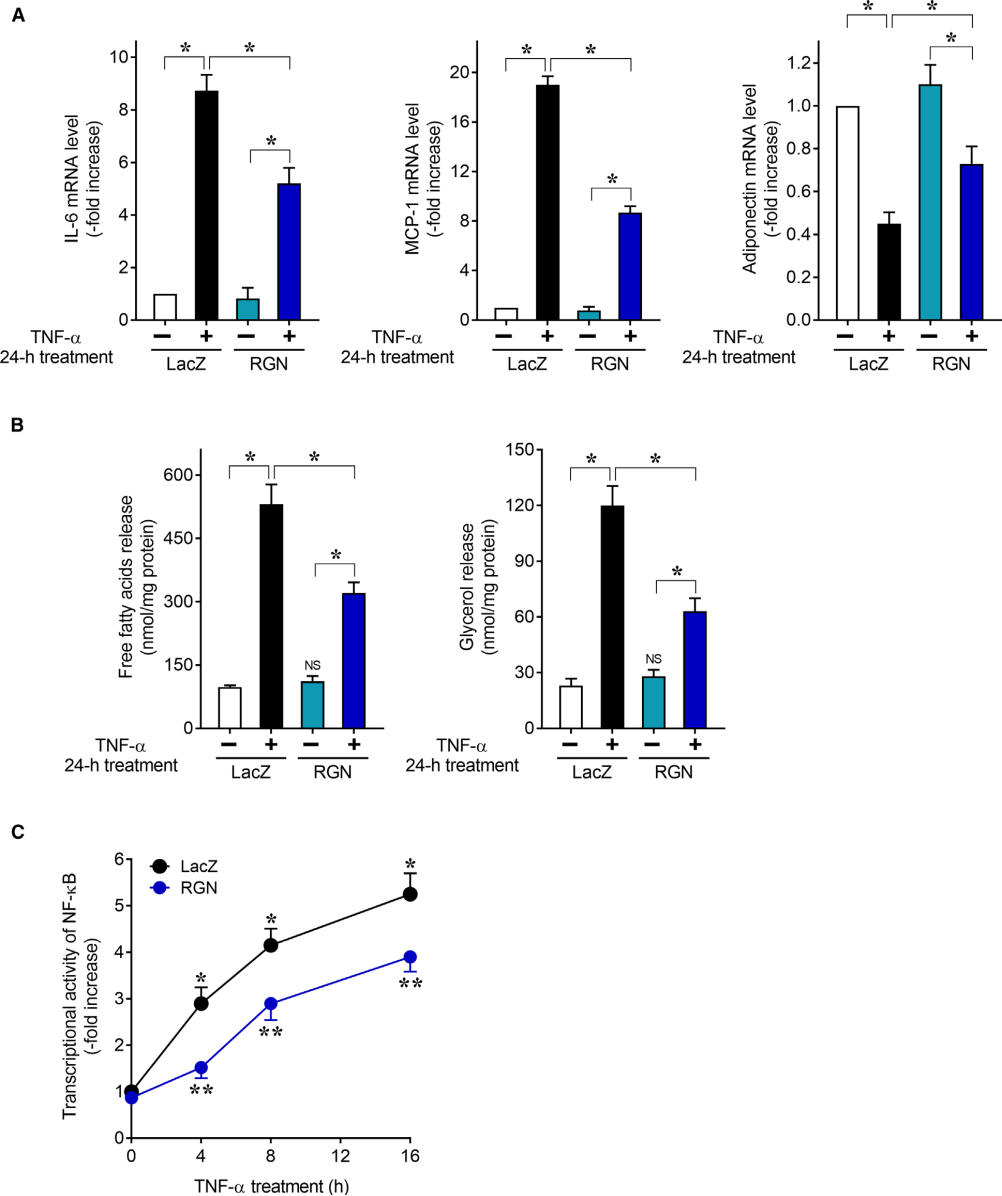
Regucalcin overexpression inhibited TNF‐α‐induced inflammatory gene expression and lipolysis in 3T3‐L1 adipocytes. (A) After 8 days of differentiation induction, 3T3‐L1 adipocytes were infected with an adenovirus harboring expression of LacZ or RGN, and 48 h later, the infected cells were treated with or without TNF‐α for 24 h. After treatment with TNF‐α, total mRNA was isolated and then subjected to real‐time RT‐PCR analysis using primer pairs for IL‐6, MCP‐1, and adiponectin mRNA. Data are presented as fold increases compared with untreated LacZ‐overexpressing cells, which were set at 1.0. Data are presented as means ± SE of three independent experiments performed in triplicate, *P < 0.05 was considered statistically significant. (B) After 8 days of differentiation induction, 3T3‐L1 adipocytes were infected with an adenovirus harboring expression of LacZ or RGN, and 48 h later, the infected adipocytes were serum‐starved for 12 h and then treated with or without TNF‐α for 24 h. The culture medium was collected and assayed for FFAs and glycerol content. Data are presented as means ± SE of three independent experiments performed in triplicate, *P < 0.05 was considered statistically significant. ‘NS’ represents ‘not significant’: untreated RGN‐overexpressing cells versus untreated LacZ‐overexpressing cells. (C) After 8 days of differentiation induction, 3T3‐L1 adipocytes stably transfected with NF‐κB‐responsive reporter vector (NF‐κB‐3T3‐L1 adipocytes) were infected with an adenovirus harboring expression of LacZ or RGN. After 48 h, the infected cells were treated with or without TNF‐α for the indicated periods and then lysed for NF‐κB reporter assay. Data are presented as fold increases compared with untreated LacZ‐overexpressing cells, which were set at 1.0. Data are presented as means ± SE of three independent experiments performed in triplicate. *P < 0.05, TNF‐α‐treated LacZ‐overexpressing cells versus untreated LacZ‐overexpressing cells at 0 time; **P < 0.05, TNF‐α‐treated RGN‐overexpressing cells versus TNF‐α‐treated LacZ‐overexpressing cells at the same time point. All data were statistically analyzed using the Kruskal–Wallis and Mann–Whitney *U*‐tests.

Macrophage‐mediated adipocyte inflammation leads to obesity and associated disorders [21]. There is evidence of crosstalk between macrophages and adipocyte via proinflammatory cytokines and chemokines in the microenvironment of obese adipose tissue [37, 38, 39]. The inflammatory communication between macrophages and adipocytes is mediated by various factors such as TNF‐α [22], MCP‐1 [24], and IL‐6 [24]. TNF‐α from adipose tissue macrophages contributes to inflammation and insulin resistance in adipocytes [23]. Furthermore, in adipocytes, TNF‐α can upregulate production of proinflammatory cytokine IL‐6 and chemokine MCP‐1 [28] and downregulate production of adiponectin [27]. To mimic chronic low‐grade inflammation of adipose tissue, we first employed TNF‐α‐mediated 3T3‐L1 adipocyte inflammation experiments. We transduced exogenous RGN into differentiated 3T3‐L1 adipocytes by the adenovirus‐mediated gene transfer method and examined the effect of RGN overexpression on TNF‐α‐induced changes in inflammatory gene expression (Fig. 3A). We confirmed that TNF‐α increased IL‐6 and MCP‐1 mRNA expression and decreased adiponectin mRNA expression in control LacZ‐overexpressing 3T3‐L1 adipocytes. Interestingly, RGN‐overexpressing 3T3‐L1 adipocytes showed a significant decrease in TNF‐α‐induced upregulation of IL‐6 and MCP‐1 and downregulation of adiponectin as compared to LacZ‐overexpressing 3T3‐L1 adipocytes. These results suggest that RGN overexpression in 3T3‐L1 adipocytes attenuated TNF‐α‐induced changes in expression of IL‐6, MCP‐1, and adiponectin.

TNF‐α is also known to dysregulate lipolysis in adipose tissues, leading to promotion of lipolysis in the adipocytes and subsequent high levels of circulating FFAs in obesity states [23]. We examined the effect of RGN overexpression on the TNF‐α‐induced hydrolysis of triglycerols into FFAs and glycerol in 3T3‐L1 adipocytes (Fig. 3B). Treatment with LacZ‐overexpressing 3T3‐L1 adipocytes with TNF‐α showed a higher release of FFAs and glycerol into the culture medium as compared to untreated LacZ‐overexpressing 3T3‐L1 adipocytes. In the nontreatment condition, there was no significant difference in the release of FFAs and glycerol into the culture medium between LacZ overexpression and RGN overexpression, implying that RGN overexpression itself does not affect lipolysis in the 3T3‐L1 adipocytes. However, following TNF‐α treatment, RGN overexpression in the 3T3‐L1 adipocytes resulted in a decrease in the release of FFAs and glycerol into the culture medium as compared to LacZ overexpression. These results suggest that RGN overexpression attenuates TNF‐α‐induced lipolysis in 3T3‐L1 adipocytes.

In adipocytes, TNF‐α activates transcriptional factor NF‐κB to induce expression of proinflammatory cytokines such as IL‐6 and MCP‐1 [40, 41]. In addition, NF‐κB activation is essential for TNF‐α‐induced lipolysis [42]. We examined the effects of RGN overexpression on NF‐κB activation in TNF‐α‐treated 3T3‐L1 adipocytes (Fig. 3C). RGN‐overexpressing 3T3‐L1 adipocytes showed decreased NF‐κB activity after TNF‐α treatment as compared to LacZ‐overexpressing 3T3‐L1 adipocytes. These results suggest an inhibitory effect of RGN overexpression on TNF‐α‐induced upregulation of IL‐6 and MCP‐1 (Fig. 3A) and enhancement of triglycerol hydrolysis (Fig. 3B) owing to attenuation of NF‐κB activation. RGN might have suppressed TNF‐α‐induced proinflammatory cytokine expression and lipolysis by attenuating NF‐κB activation. It has been reported that RGN attenuates oxidative stress‐induced NF‐κB activation by maintaining a balance between protein tyrosine kinase and protein tyrosine phosphatase in prostate endothelial cells [43]. Because TNF‐α is known to cause oxidative stress in adipocytes [44], it is important to investigate whether RGN can inhibit TNF‐α‐induced inflammatory response gene expression and lipolysis by preventing the imbalance between protein tyrosine kinase and protein tyrosine phosphatase in 3T3‐L1 adipocytes.

**Fig. 4:**
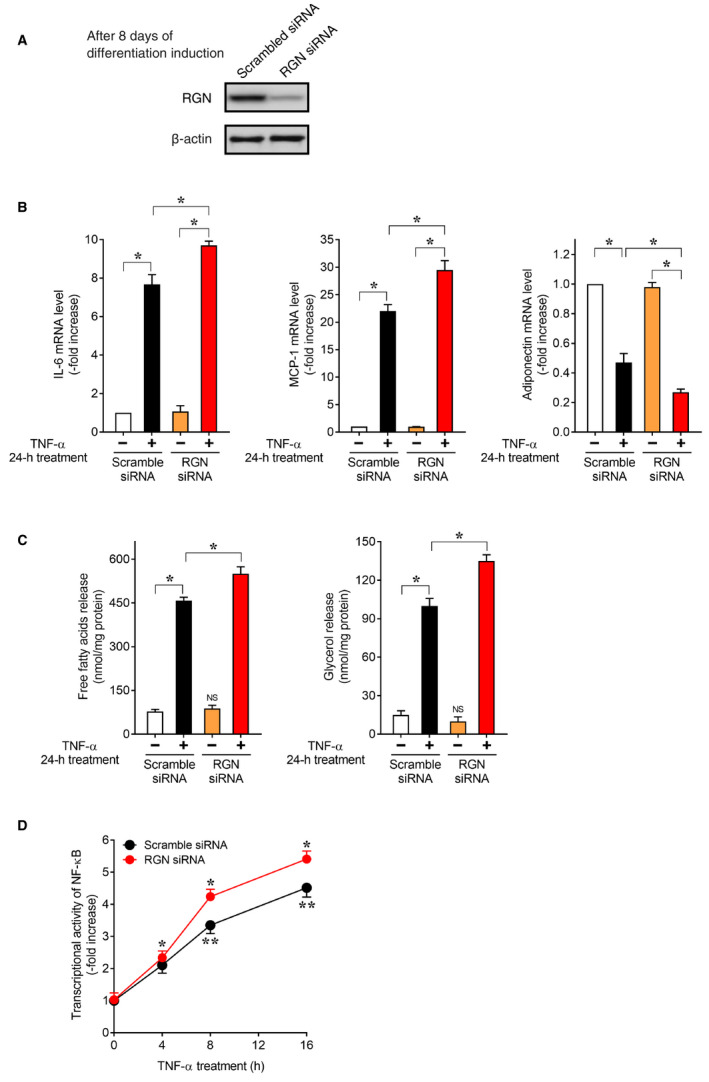
Regucalcin knockdown enhanced TNF‐α‐induced inflammatory gene expression and lipolysis in mature 3T3‐L1 adipocytes. After 8 days of differentiation induction, 3T3‐L1 adipocytes were transfected with scrambled control siRNA and RGN‐targeting siRNA. (A) After 48 h of siRNA transfection, the knockdown efficiency of RGN was examined by western blotting using anti‐RGN antibody. β‐Actin was used as a loading control. (B) After 48 h of siRNA transfection, cells were treated with or without TNF‐α for 24 h. Total mRNA was isolated and then subjected to real‐time RT‐PCR analysis using primer pairs for IL‐6, MCP‐1, and adiponectin mRNA. Data are presented as fold increases compared with untreated scrambled siRNA‐transfected cells, which were set at 1.0. Data are presented as means ± SE of three independent experiments performed in triplicate, *P < 0.05 was considered statistically significant. (C) After 48 h of siRNA transfection, cells were serum‐starved for 12 h and then treated with or without TNF‐α for 24 h. The culture medium was collected and assayed for FFAs and glycerol content. Data are presented as means ± SE of three independent experiments performed in triplicate, *P < 0.05 was considered statistically significant. ‘NS’ represents ‘not significant’: untreated RGN‐targeting siRNA‐transfected cells versus untreated scrambled siRNA‐transfected cells. (D) After 8 days of differentiation induction, the NF‐κB‐3T3‐L1 adipocytes were transfected with scrambled control siRNA and RGN‐targeting siRNA. After 48 h of transfection, cells were treated with or without TNF‐α for the indicated periods and then lysed for NF‐κB reporter assay. Data are presented as fold increases compared with untreated scrambled siRNA‐transfected cells, which were set at 1.0. Data are presented as means ± SE of three independent experiments performed in triplicate, *P < 0.05 was considered statistically significant. *P < 0.05, TNF‐α‐treated scrambled siRNA‐transfected cells versus untreated scrambled siRNA‐transfected cells at 0 time; **P < 0.05, TNF‐α‐treated RGN siRNA‐transfected cells versus TNF‐α‐treated scrambled siRNA‐transfected cells at the same time point. All data were statistically analyzed using the Kruskal–Wallis and Mann–Whitney *U*‐tests.

To further confirm the inhibitory effect of RGN on TNF‐α‐induced inflammatory response in 3T3‐L1 adipocytes, we examined the siRNA‐mediated knockdown effect of RGN on TNF‐α‐induced inflammation in 3T3‐L1 adipocytes. As shown in Fig. 4A, immunoblotting analysis showed that transfection with RGN‐targeting siRNA silenced endogenous RGN protein expression in 3T3‐L1 adipocytes relative to scrambled siRNA transfection. As shown in Fig. 4B, following TNF‐α treatment, RGN siRNA‐transfected 3T3‐L1 adipocytes showed increased expression of IL‐6 and MCP‐1 mRNA and decreased expression of adiponectin mRNA as compared to scrambled siRNA‐transfected control cells, indicating that RGN silencing enhanced the effectiveness of TNF‐α against expression of IL‐6, MCP‐1, and adiponectin. In addition, as shown in Fig. 4C, in the nontreatment condition, there was no significant difference in the release of FFAs and glycerol into the culture medium between the scrambled siRNA‐transfected 3T3‐L1 adipocytes and RGN siRNA‐transfected 3T3‐L1 adipocytes, implying that RGN silencing does not affect lipolysis in the 3T3‐L1 adipocytes. However, following TNF‐α treatment, RGN siRNA‐transfected 3T3‐L1 adipocytes exhibited a significant increase in the release of FFAs and glycerol into the culture medium as compared to scrambled siRNA‐transfected cells, indicating that RGN silencing enhanced TNF‐α‐induced lipolysis. Furthermore, as shown in Fig. 4D, upon treatment with TNF‐α, RGN siRNA‐transfected 3T3‐L1 adipocytes had significantly higher NF‐κB transcriptional activity as compared to scrambled siRNA‐transfected cells, indicating that RGN silencing enhanced TNF‐α‐induced NF‐κB transcriptional activity. Consistent with the RGN overexpression experiment data, the RGN knockdown experiment further showed a suppressive effect of RGN on TNF‐α‐induced inflammation and lipolysis via NF‐κB activation in 3T3‐L1 adipocytes.

### RGN blocks inflammatory changes in interaction between 3T3‐L1 adipocytes and RAW264.7 macrophages under coculture conditions

**Fig. 5:**
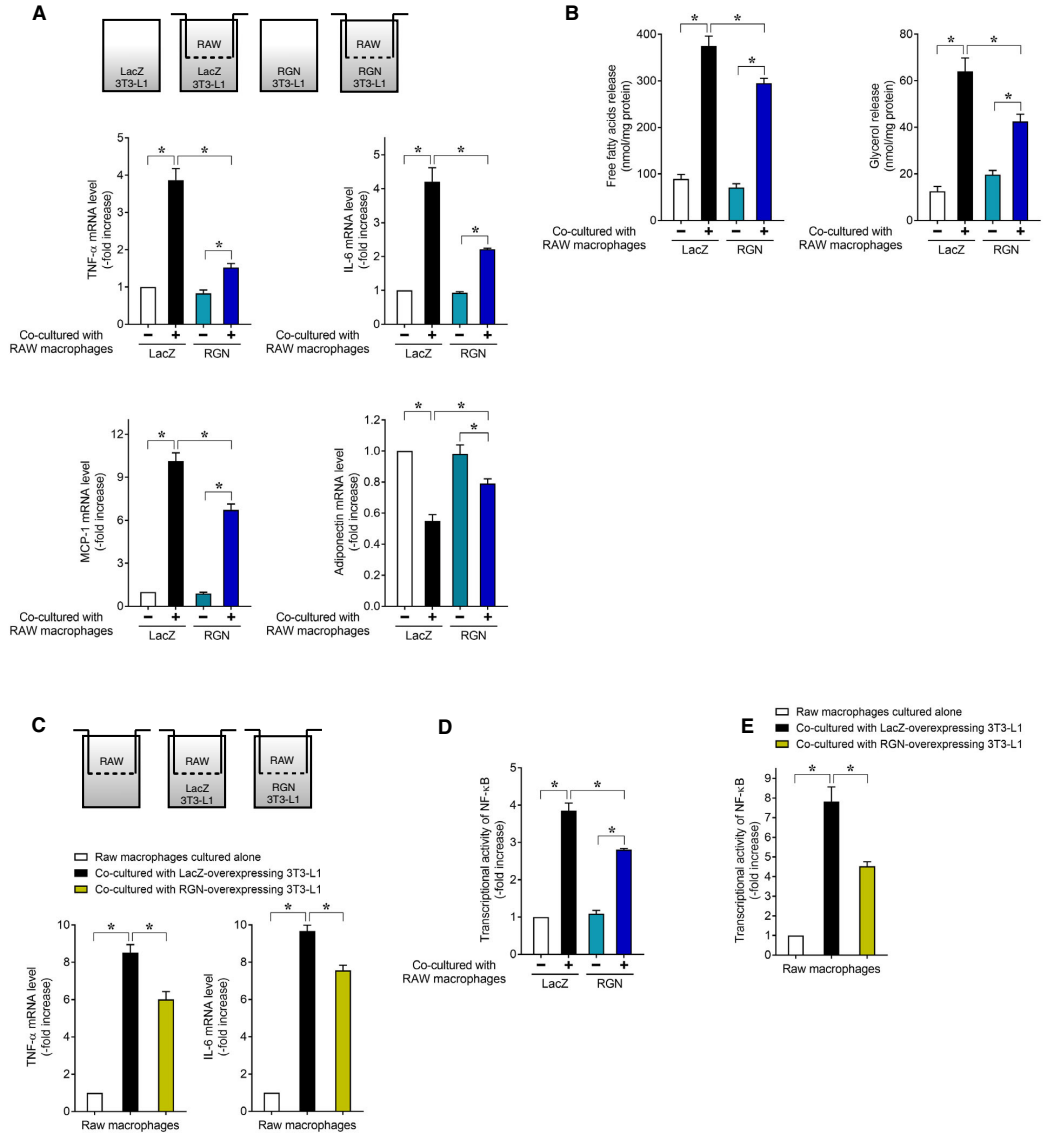
Regucalcin overexpression in 3T3‐L1 adipocytes blocked inflammatory communication between 3T3‐L1 adipocytes and RAW264.7 macrophages in coculture systems. After 8 days of differentiation induction, 3T3‐L1 adipocytes or NF‐κB‐3T3‐L1 adipocytes were infected with an adenovirus harboring expression of LacZ or RGN. Then, 48 h later, infected cells were used for the subsequent experiments. (A) The adenovirus‐infected 3T3‐L1 adipocytes were cocultured with or without RAW264.7 macrophages for 24 h. Thereafter, the total mRNA of 3T3‐L1 adipocytes was isolated and then subjected to real‐time RT‐PCR analysis using primer pairs for TNF‐α, IL‐6, MCP‐1, and adiponectin mRNA. Data are presented as fold increases compared with single culture of 3T3‐L1 adipocytes overexpressing LacZ, which were set at 1.0. Data are presented as means ± SE of three independent experiments performed in triplicate, *P < 0.05 was considered statistically significant. (B) The adenovirus‐infected 3T3‐L1 adipocytes were serum‐starved for 12 h and then cocultured with or without RAW264.7 macrophages for 24 h. The culture medium was collected and assayed for FFAs and glycerol content. Data are presented as means ± SE of three independent experiments performed in triplicate, *P < 0.05 was considered statistically significant. (C) RAW264.7 macrophages were cocultured with or without adenovirus‐infected 3T3‐L1 adipocytes for 24 h, and the total mRNA of RAW264.7 macrophages was then processed for real‐time RT‐PCR analysis using primer pairs for TNF‐α and IL‐6. Data are presented as fold increases compared with RAW264.7 macrophages cultured alone, which was set at 1.0. Data are presented as means ± SE of three independent experiments performed in triplicate, *P < 0.05 was considered statistically significant. (D) The adenovirus‐infected NF‐κB‐3T3‐L1 adipocytes were cocultured with or without RAW264.7 macrophages for 16 h, and the cell lysates of the NF‐κB‐3T3‐L1 adipocytes were used for NF‐κB reporter assay. Data are presented as fold increases compared with single culture of NF‐κB‐3T3‐L1 adipocytes overexpressing LacZ, which were set at 1.0. Data are presented as means ± SE of three independent experiments performed in triplicate, *P < 0.05 was considered statistically significant. (E) RAW264.7 macrophages stably transfected with NF‐κB‐responsive reporter vector (NF‐κB‐RAW264.7 macrophages) were cocultured with or without adenovirus‐infected 3T3‐L1 adipocytes for 16 h, and the cell lysate of the NF‐κB‐RAW264.7 macrophages was used for NF‐κB reporter assay. Data are presented as fold increases compared with NF‐κB‐RAW264.7 macrophages cultured alone, which were set at 1.0. Data are presented as means ± SE of three independent experiments performed in triplicate, *P < 0.05 was considered statistically significant. All data were statistically analyzed using the Kruskal–Wallis and Mann–Whitney *U*‐tests.

An *in vitro* coculture experiment using 3T3‐L1 adipocytes and RAW264.7 macrophages was established to estimate crosstalk between macrophages and adipocytes in macrophage‐infiltrate obese adipocyte tissue [28, 29]. We transduced the RGN gene into 3T3‐L1 adipocytes using an adenovirus‐mediated gene transfer system and then employed a coculture system with RGN‐overexpressing 3T3‐L1 adipocytes and RAW264.7 macrophages. As shown in Fig. 5A, LacZ‐overexpressing 3T3‐L1 adipocytes cocultured with RAW264.7 macrophages showed a significant increase in mRNA expression of TNF‐α, IL‐6, and MCP‐1 and a significant decrease in mRNA expression of adiponectin as compared to culture of LacZ‐overexpressing 3T3‐L1 adipocytes alone, indicating the existence of a paracrine effect of RAW264.7 macrophages on inflammatory gene expression in 3T3‐L1 adipocytes. In contrast, RGN overexpression in 3T3‐L1 adipocytes attenuated changes in expression of TNF‐α, IL‐6, MCP‐1, and adiponectin after coculture with RAW264.7 macrophages relative to LacZ overexpression. These results suggest that RGN overexpression in 3T3‐L1 adipocytes blocked the paracrine effect of RAW264.7 macrophages on inflammatory gene expression in 3T3‐L1 adipocytes.

Previously established *in vitro* coculture studies have shown that in the paracrine loop between 3T3‐L1 adipocytes and RAW264.7 macrophages, FFAs released from 3T3‐L1 adipocytes act as a paracrine mediator against RAW264.7 macrophages and subsequently cause inflammation in RAW264.7 macrophages [28, 29]. We investigated whether RGN could block the incidence of an FFA‐mediated paracrine effect of 3T3‐L1 adipocytes on RAW264.7 macrophage inflammation. As shown in Fig. 5B, we first confirmed that coculturing LacZ‐overexpressing 3T3‐L1 adipocytes with RAW264.7 macrophages results in a significant increase in release of FFAs and glycerol into the coculture medium as compared to LacZ‐overexpressing 3T3‐L1 adipocytes cultured alone. Interestingly, after coculture with RAW264.7 macrophages, RGN overexpression in 3T3‐L1 adipocytes exhibited decreased release of FFAs and glycerol into the coculture medium relative to LacZ‐overexpressing 3T3‐L1 adipocytes, indicating an inhibitory effect of RGN overexpression on coculture‐induced lipolysis in 3T3‐L1 adipocytes. In addition, as shown in Fig. 5C, when RAW264.7 macrophages were used for coculture of LacZ‐overexpressing 3T3‐L1 adipocytes, the mRNA expression of TNF‐α and IL‐6 was significantly increased in RAW264.7 macrophages as compared to RAW264.7 macrophages cultured alone, indicating the existence of a paracrine effect of 3T3‐L1 adipocytes on inflammatory gene expression in RAW264.7 macrophages. In contrast, the RAW264.7 macrophages used for coculture of RGN‐overexpressing 3T3‐L1 adipocytes had significantly lower mRNA expression of TNF‐α and IL‐6 as compared to the RAW264.7 macrophages used for LacZ‐overexpressing 3T3‐L1 adipocyte coculture. Considering the inhibitory effect of RGN overexpression on increased lipolysis in cocultured 3T3‐L1 adipocytes (Fig. 5B), these results suggest that in the coculture system, RGN overexpression in 3T3‐L1 adipocyte blocked the incidence of an FFA‐mediated paracrine effect of 3T3‐L1 adipocytes on the upregulation of two proinflammatory genes in RAW264.7 macrophages.

Previously established *in vitro* coculture studies have reported that (a) the NF‐κB pathway is activated in both 3T3‐L1 adipocytes and RAW264.7 macrophages via paracrine interaction between the two cell types, (b) NF‐κB activation in cocultured 3T3‐L1 adipocytes contributes to FFAs release, and (c) the FFAs, as 3T3‐L1 adipocyte‐derived paracrine mediators, cause NF‐κB activation‐mediated TNF‐α production in RAW264.7 macrophages [28, 29]. As shown in Fig. 5D, significantly decreased NF‐κB activity was observed in cocultured 3T3‐L1 adipocytes overexpressing RGN relative to cocultured 3T3‐L1 adipocytes overexpressing LacZ. In addition, as shown in Fig. 5E, coculture of RGN‐overexpressing 3T3‐L1 adipocytes led to a decrease in NF‐κB activity in RAW264.7 macrophages. Considering the inhibitory effect of RGN overexpression on lipolysis in cocultured 3T3‐L1 adipocytes (Fig. 5B), these results suggest that under coculture conditions, the blocking of NF‐κB activation in 3T3‐L1 adipocytes by RGN overexpression caused a decrease in release of FFAs, and this led to a decrease in FFA‐induced NF‐κB activation in RAW264.7 macrophages, accompanied by decreased upregulation of TNF‐α and IL‐6 (Fig. 5C).

**Fig. 6:**
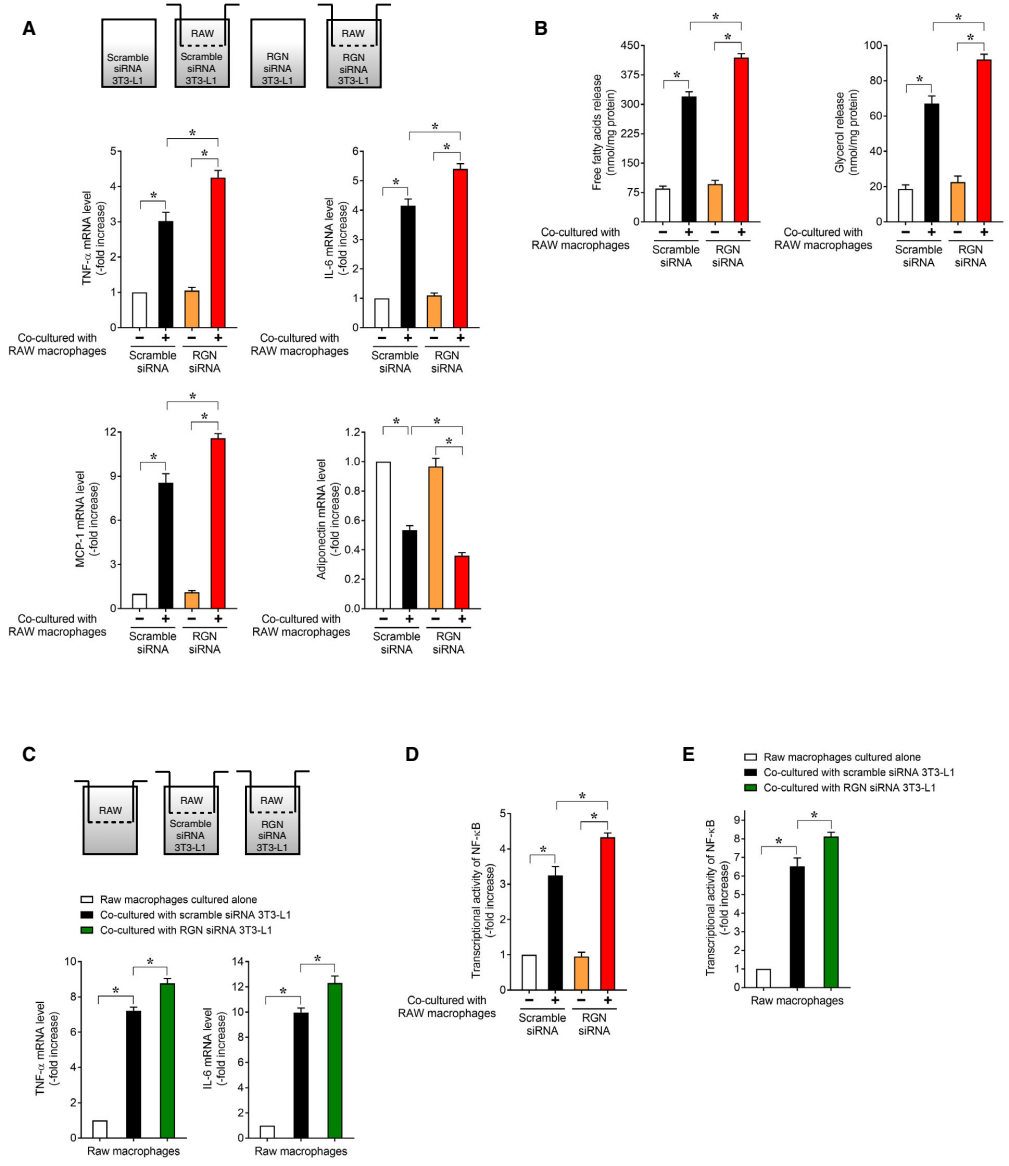
Regucalcin knockdown in 3T3‐L1 adipocytes promoted inflammatory communication between 3T3‐L1 adipocytes and RAW264.7 macrophages in coculture systems. After 8 days of differentiation induction, 3T3‐L1 adipocytes or NF‐κB‐3T3‐L1 adipocytes were transfected with scrambled control siRNA and RGN‐targeting siRNA. After 48 h, the transfected cells were used for the subsequent experiments. (A) The transfected 3T3‐L1 adipocytes were cocultured with or without RAW264.7 macrophages for 24 h. Thereafter, the total mRNA of transfected 3T3‐L1 adipocytes was extracted and processed for real‐time RT‐PCR analysis using primer pairs for TNF‐α, IL‐6, MCP‐1, and adiponectin mRNA. Data are presented as fold increases compared with single culture of scrambled siRNA‐transfected 3T3‐L1 adipocytes, which were set at 1.0. Data are presented as means ± SE of three independent experiments performed in triplicate, *P < 0.05 was considered statistically significant. (B) The transfected 3T3‐L1 adipocytes were serum‐starved for 12 h and then cocultured with or without RAW264.7 macrophages for 24 h. The culture medium was collected and assayed for FFAs and glycerol content. Data are presented as means ± SE of three independent experiments performed in triplicate, *P < 0.05 was considered statistically significant. (C) RAW264.7 macrophages were cocultured with or without transfected 3T3‐L1 adipocytes for 24 h, and the total mRNA of RAW264.7 macrophages was isolated and then processed for real‐time RT‐PCR analysis using primer pairs for TNF‐α and IL‐6. Data are presented as fold increases compared with RAW264.7 macrophages cultured alone, which were set at 1.0. Data are presented as means ± SE of three independent experiments performed in triplicate, *P < 0.05 was considered statistically significant. (D)The transfected NF‐κB‐3T3‐L1 adipocytes were cocultured with or without RAW264.7 macrophages for 16 h, and cell lysates of the transfected NF‐κB‐3T3‐L1 adipocytes were used for NF‐κB reporter assay. Data are presented as fold increases compared with single culture of scrambled siRNA‐transfected NF‐κB‐3T3‐L1 adipocytes, which were set at 1.0. Data are presented as means ± SE of three independent experiments performed in triplicate, *P < 0.05 was considered statistically significant. (E) The transfected 3T3‐L1 adipocytes were cocultured with NF‐κB‐RAW264.7 macrophages for 16 h, and cell lysates of the NF‐κB‐RAW264.7 macrophages were used for NF‐κB reporter assay. Data are presented as fold increases compared with NF‐κB‐RAW264.7 macrophages cultured alone, which were set at 1.0. Data are presented as means ± SE of three independent experiments performed in triplicate, *P < 0.05 was considered statistically significant. All data were statistically analyzed using the Kruskal–Wallis and Mann–Whitney *U*‐tests.

We further explored whether siRNA‐mediated RGN knockdown of 3T3‐L1 adipocytes could disturb the paracrine loop between 3T3‐L1 adipocytes and RAW264.7 macrophages in a coculture system. As shown in Fig. 6A, following coculture with RAW264.7 macrophages, control 3T3‐L1 adipocytes transfected with scrambled siRNA showed increased mRNA expression of TNF‐α, IL‐6, and MCP‐1 and decreased mRNA expression of adiponectin. In contrast, when RAW264.7 macrophages were present, transfection of RGN‐targeting siRNA into 3T3‐L1 adipocytes led to significantly higher upregulation of TNF‐α, IL‐6, and MCP‐1 expression and lower downregulation of adiponectin relative to transfection with scrambled siRNA, indicating that RGN silencing of 3T3‐L1 adipocytes augmented coculture‐induced changes in TNF‐α, IL‐6, MCP‐1, and adiponectin expression. These results suggest that under coculture conditions, the paracrine effect of RAW264.7 macrophages on inflammatory gene expression in 3T3‐L1 adipocytes was enhanced by RGN silencing of 3T3‐L1 adipocytes.

As shown in Fig. 6B, following coculture with RAW264.7 macrophages, the release of FFAs and glycerol into the culture medium was significantly higher in the coculture of RGN siRNA‐transfected 3T3‐L1 adipocytes than with scrambled siRNA‐transfected adipocytes, indicating the enhancement of FFA release from RGN‐silenced 3T3‐L1 adipocytes. Additionally, as shown in Fig. 6C, significantly increased mRNA expression of TNF‐α and IL‐6 mRNA was observed in the RAW264.7 macrophages used for coculture of RGN siRNA‐transfected 3T3‐L1 adipocytes as compared to the RAW264.7 macrophages used for scrambled siRNA‐transfected 3T3‐L1 adipocyte coculture. These results suggest that the increased release of FFAs from RGN‐silenced 3T3‐L1 adipocytes led to upregulation of two proinflammatory genes in RAW264.7 macrophages.

Furthermore, as shown in Fig. 6D,E, coculture of RGN‐targeting siRNA‐transfected 3T3‐L1 adipocytes with RAW264.7 macrophages resulted in a significant increase in NF‐κB activity in both 3T3‐L1 adipocytes with RAW264.7 macrophages as compared to coculture of scrambled siRNA‐transfected 3T3‐L1 adipocytes, indicating that RGN silencing of 3T3‐L1 adipocytes enhanced NF‐κB activation in both 3T3‐L1 adipocytes and RAW264.7 macrophages. These results indicate that higher NF‐κB activation in 3T3‐L1 adipocytes by RGN silencing enhanced FFA release and that subsequent FFA‐mediated NF‐κB activation in RAW264.7 macrophages resulted in the upregulation of proinflammatory genes. Consistent with the RGN overexpression experiment, the RGN knockdown experiments indicated that RGN could act as an anti‐inflammatory factor in 3T3‐L1 adipocytes and block inflammatory communication between 3T3‐L1 adipocytes and RAW264.7 macrophages under the coculture conditions.

Using a coculture system with 3T3‐L1 adipocytes and RAW264.7 macrophages, we found that RGN in 3T3‐L1 adipocytes blocked the paracrine effect of RAW264.7 macrophages on inflammation in 3T3‐L1 adipocytes and then prevented the incidence of a paracrine effect of 3T3‐L1 adipocytes on inflammation in RAW264.7 macrophages. These findings demonstrate that RGN could ameliorate inflammatory responses resulting from the paracrine interaction of 3T3‐L1 adipocytes and RAW264.7 macrophages. Thus, RGN might exert anti‐inflammatory effects on macrophage‐facilitated obese adipocyte tissue, and our findings provide a novel insight into an adipocyte defense mechanism against inflammatory communication between adipocytes and macrophages in macrophage‐infiltrated obese adipocyte tissues. We found that the expression of RGN protein was transiently elevated in the subcutaneous adipose tissues when a high‐fat diet was fed to early‐aged mice as compared to the expression level when a normal diet was fed, and the high‐fat diet obesity mice showed a decreased RGN expression in the subcutaneous adipose tissue with aging (TM and MY, unpublished data), suggesting that a change in the RGN expression contributes to the onset and progression of obesity. Further *in vivo* investigation of the anti‐inflammatory effect of RGN on adipocyte inflammation could lead to a better understanding of the pathogenic mechanism of obesity and related disorders.

## Conclusion

In the present study, we found that RGN enhanced the differentiation of 3T3‐L1 cells into adipocytes. This finding provides a new adipocyte differentiation mechanism and indicates that RGN might be a therapeutic target for abnormally enhanced adipogenesis in obesity. In addition, we found that RGN attenuated TNF‐α‐induced inflammation in 3T3‐L1 adipocytes and disturbed the paracrine loop between 3T3‐L1 adipocytes and RAW264.7 macrophages under coculture conditions. These findings demonstrate that RGN might be a therapeutic target for preventing macrophage‐associated adipocyte inflammation.

## Conflict of interest

The authors declare no conflict of interest.

## Author contributions

TM, SK, RW, KH, and KH performed the experiments. SK and CT constructed retrovirus expression system. TM and MY designed the experiment and discussed with NK. MY provided comments pertaining to the manuscript. TM wrote the manuscript. All authors read and commented on this manuscript.

## Data Availability

The data that support the findings of this study are available from the corresponding author, TM, upon reasonable request.
